# *Mycoplasma Genitalium* and *Mycoplasma Hominis* are prevalent and correlated with HIV risk in MSM: a cross-sectional study in Shenyang, China

**DOI:** 10.1186/s12879-019-4138-5

**Published:** 2019-06-04

**Authors:** Ning Zhao, Katherine T. Li, Yang-yang Gao, Jun-jie Xu, De-Sheng Huang

**Affiliations:** 1 Department of Epidemiology, School of Public Health, China Medical University, Shenyang, China; 2 Weill Cornell Medical College, New York, NY USA; 3 Key Laboratory of AIDS Immunology of National Health Commission, Department of Laboratory Medicine, The First Affiliated Hospital, China Medical University, Shenyang, 110001 Liaoning China; 4 Department of Mathematics, School of Fundamental Sciences, China Medical University, Shenyang, 110122 China; 5 Department of Ophthalmology, The First Affiliated Hospital of China Medical University, Shenyang, China

**Keywords:** Men who have sex with men, Sexually transmitted infection, Mycoplasma genitalium, Mycoplasma hominis, Syphilis, HIV, Geosocial networking apps

## Abstract

**Background:**

A high proportion of men who have sex with men (MSM) use geosocial networking apps (Apps) to seek partners. However, the relationship of app use with HIV risk is unknown. Further, the risks of some sexually transmitted infection (STIs), including *Mycoplasma genitalium,* have seldom been studied among MSM.

**Methods:**

MSM were enrolled at a community-based HIV testing site in Shenyang, China. After completing a questionnaire survey, we collected rectal swabs and venous blood specimens. We then simultaneously tested for ten STIs *(Chlamydia trachomatis* [CT], *Neisseria gonorrhea* [NG], *Ureaplasma urealyticum* [Uu], *Ureaplasma parvum* species [Up1, Up3, Up6, Up14), *Mycoplasma hominis* [Mh], *Mycoplasma genitalium* [Mg], and Herpes Simplex Virus Type 2 (HSV-2) using multiple PCR. We also performed blood tests for HIV, Syphilis, Hepatitis C antibody (HCV-Ab), Hepatitis B Surface Antigen (HBsAg), and Hepatitis A-IgM (HAV-IgM), etc.

**Results:**

One hundred and eighty-three MSM participated in this study, of which 51.4% reported seeking partners through apps in the past year. The prevalence of HIV was 19.7%, Syphilis 12.0%, HAV 1.1%, rectal Mg 15.3% and Mh 7.1%. Multivariable logistic regression showed that HIV infection was independently correlated with app-using behavior (adjusted odds ratio[aOR] = 2.6), Mg infection (aOR = 3.2), Mh infection (aOR = 4.1) and Syphilis infection (aOR = 3.1) (each *P* < 0.05).

**Conclusions:**

App use, Mg, Mh and Syphilis infection were correlated with higher HIV Risk in MSM. Geosocial networking apps should be utilized for HIV interventions targeting MSM. There is a need for more expansive STIs screening, particularly for Mg, Mh and Syphilis in MSM.

## Background

There is a growing HIV epidemic among Chinese MSM. The number of new reported HIV/AIDS cases transmitted through homosexual sex increased from 26,746 in 2014 [1] to 34,358 in 2018 [2], with an average annual increase of 9.5%. It is urgent to determine the factors associated with HIV infection in order to design targeted intervention strategies to control the spread of HIV among MSM.

One area of investigation is the recent shift among MSM communities from offline to online methods of seeking sexual partners. Two recent prospective studies (including one from China) found that seeking sexual partners through geosocial networking apps (apps) was correlated with elevated HIV incidence in MSM [3, 4]. However, another recent meta-analysis failed to find an association between app use and HIV infection, there was an association with higher rates of other STIs [5]. Thus, more studies are needed to better understand the relationship between app use and HIV risk.

Sexually transmitted infections (STIs) are another important risk factor for HIV infection in MSM [6]. STIs traditionally monitored among Chinese MSM include *Chlamydia, Gon*orrhoea, Hepatitis B virus (HBV), Hepatitis C virus (HCV) and Syphilis [7]. There is limited data on many other STIs, including Hepatitis A virus (HAV) and *Mycoplasma* species. HAV can spread through oral-anal intercourse and digital-rectal intercourse, and is at high risk during group sex [8]. Recently there have been multiple outbreak reports of HAV in MSM in Western countries, including Australia [9], Italy [10], England [11] and Netherlands [12]. Additionally, mycoplasma species (including both *Mycoplasma genitalium* [Mg] *and Mycoplasma hominis* [Mh]) can cause urethritis. Two studies found an association between Mg infection and HIV in MSM [13, 14], but one other study failed to find a significant association [15]. Testing for Mg is limited among MSM [16], and very few studies report the prevalence of Mg and Mh in MSM [13, 14, 17–21]. As a result, the relationships of Mg and Mh infection with HIV are not well established among MSM.

To address these gaps in knowledge regarding app use and STI infection and their relationship with HIV risk, we performed a cross-sectional study in Shenyang, China to 1) study the app-using behaviors of Chinese MSM and the relationship between app usage and HIV; and 2) determine the prevalence of traditionally monitored STIs (including Chlamydia, Gonorrhoea, Hepatitis B Surface Antigen [HBsAg], HCV-Ab and Syphilis) and neglected STIs, (including HAV, Mg and Mh, etc.) among MSM, and investigate the association between these STIs and HIV infection.

## Methods

### Recruitment and data collection

During March 2018 to October 2018, we recruited 183 MSM who sought voluntary HIV counseling and testing at the First Affiliated Hospital of China Medical University (CMU) in Shenyang, China. Eligibility criteria included (1) being at least 18 years old, (2) being biologically male; (3) self-reported anal or oral sex with another male in the past 12 months, (4) being able to provide written informed consent.

After informed consent, each participant was asked to complete an anonymous self-filled e-questionnaire [3]. We collected data on age, occupation, monthly salary, number of male sexual partners, condom use with male sexual partners, preferred sexual roles, STIs in the past 12 months, and partner-seeking methods.

Approximately 10 ml of blood were drawn by a trained nurse. Participants then self-collected rectal swab samples for HIV and STIs testing. Unique identification numbers were used to match the e-questionnaire and laboratory test results.

### Collection and testing of rectal specimens

Trained staff counseled participants on how to collect one-time rectal swabs for rectal exfoliated cells and secretions 2–3 cm inside the anus. We processed rectal swab sample DNA and used biotin-labeled multiple specific primers for *Chlamydia* [CT], *Neisseria Gonorrhea* [NG], *Ureaplasma urealyticum* [Uu], *Ureaplasma parvum* species [Up1, Up3, Up6, Up14], *Mycoplasma hominis* [Mh], *Mycoplasma genitalium* [Mg], and herpes simplex virus type 2 [HSV-2] fragments for PCR amplification. Amplified DNA were then hybridized with immobilized STD probes under the patented “Flow-through Hybridization” technique using the HybriMem detection kit (KaiPU Co., Ltd. Guangzhou, China). Enzyme immunoassay method was applied for color development in order to obtain test results within 1 h.

### Laboratory processing of blood samples

We tested blood samples using the HIV-HCV (Hepatitis C virus)-TP (Treponema pallidum)-HBV (Hepatitis B virus) quadruplex detection reagents (Wanfu, Guangzhou) and HAV (Hepatitis A virus) detection reagent (Wanfu, Guangzhou) to detect antibodies to HIV and Treponema pallidum antibody (TP-Ab), Hepatitis C antibody (HCV-Ab), Hepatitis B Surface Antigen (HBsAg) and Hepatitis A-IgM (HAV-IgM). HIV antibody was tested by Elecsys HIV combi PT detection kit (Roche Diagnostics GmbH, Germany), and positive tests were confirmed by HIV-1/2 Western blot assay (HIV Blot 2.2 WB; MP Biomedical Asia Pacific Pte Ltd). Syphilis was tested by rapid plasma reagent (RPR Diagnosis; Shanghai, Kehua, China), and any RPR positive samples were then confirmed via *Treponema pallidum particle assay* (TPPA Serodia, Japan). Samples that tested positive for both RPR and TPPA were considered positive for Syphilis.

### Statistical analysis

Data from the e-questionnaire and laboratory test results were analyzed using SPSS 22.0 (SPSS, Inc., Chicago, IL, USA) software. We used descriptive statistics to examine the frequency of app usage and prevalence of STIs. Chi-squared tests were used to analyze the relationships between each predictor variable and HIV infection. After adjusting for age, education and monthly economic income, we performed multivariable logistic regression analysis to obtain correlates of HIV infection reported as Odds ratios (ORs) with 95% confidence intervals (CIs).

## Results

### Sociodemographics and sexual behavior

The majority of the 183 MSM participants who completed the survey were older than 25 years (77.0%), employed full-time (76.5%), unmarried (87.4%), with middle school education or above (74.3%), and an average monthly income of ≥300 US dollars (56.3%) (Table 1).

**Table 1:** Demographic factors of MSM participants in Shenyang, China (*N* = 183)

Variables		All (%)	HIV positive (rate, %)	*P*-value
Ages (Year)	18–24	42 (23.0)	15 (35.7)	0.003
	≥25	141 (77.0)	21 (14.9)	
Occupations	Part-time or unemployed	43 (23.5)	8 (18.6)	0.869
	Full-time employed	140 (76.5)	28 (20.0)	
Schooling education	Middle school and below	47 (25.7)	10 (21.3)	0.748
	Completed middle school and above	136 (74.3)	26 (19.1)	
Marriage status	Married	23 (12.6)	4 (17.4)	0.769
	Unmarried	160 (87.4)	32 (20.0)	
Monthly income (USD, dollar)	< 300	80 (43.7)	11 (13.8)	0.076
	≥300	103 (56.3)	25 (24.3)	

More than half of participants mainly sought male sexual partners through apps (51.4%), and had sexual debut between 18 and 24 years of age (51.4%). The most common role during anal sex was versatile (39.3%). In the past 3-months, most had ≥2 male sexual partners (79.8%), and most had ≥2 casual male sexual partners (63.9%). Less than half had used condom with male sexual partners during anal intercourse (37.2%) (Table 2).

**Table 2:** Sexual behavior of MSM participants in Shenyang, China (*N* = 183)

Variables		All (%)	HIV positive (rate, %)	*P*-value
Main partner-seeking methods	Non-APPs	89 (48.6)	11 (12.4)	0.015
	APPs	94 (51.4)	25 (26.6)	
Sexual debut age (years)	< 18	52 (28.4)	11 (21.2)	0.950
	18–24	94 (51.4)	18 (19.1)	
	≥25	37 (20.2)	7 (18.9)	
Sexual roles with partners	Bottom	49 (26.8)	12 (24.5)	0.402
	Top	62 (33.9)	9 (14.5)	
	Versatile	72 (39.3)	15 (20.8)	
Male circumcision	Yes	11 (6.0)	1 (9.1)	0.362
	No	172 (94)	35 (20.3)	
Sexual behaviors in recent 3-months				
No. of total male sexual partners	0–1	37 (20.2)	5 (13.5)	0.291
	≥2	146 (79.8)	31 (21.2)	
No. of casual male sexual partners	0–1	65 (36.1)	9 (13.8)	0.141
	≥2	118 (63.9)	27 (22.9)	
Using condom with partners during anal intercourse	Always	68 (37.2)	12 (17.6)	0.684
	Occasionally	83 (45.4)	16 (19.3)	
	Never used	32 (17.5)	8 (25.0)	

### Prevalence of HIV/STIs infection

The total prevalence of HIV in MSM participants was 19.7% (36/183). The prevalence of *Mycoplasma hominis, Mycoplasma genitalium, Neisseria gonorrhoeae, Chlamydia trachomatis,* Uu, Up1, Up3, Up6, Up14 and HSV-2 were 7.1, 15.3, 0.5, 4.4, 4.4, 0.0, 0.0, 0.0, 0.0 and 1.1%, respectively. The prevalence of Syphilis was 12.0%. The prevalence of HAV, HBV, and HCV were 1.1, 3.3 and 0.5% (Table 3).

**Table 3:** Correlates of HIV infection and other STIs in MSM attendants in Shenyang, China (*N* = 183)

Variables		All (%)	HIV Positive (rate, %)	*P*-value
DNA test of 10-STIs				
Mh.	No	170 (92.9)	30 (17.6)	0.013
	Yes	13 (7.1)	6 (46.2)	
Mg.	No	155 (84.7)	25 (16.1)	0.005
	Yes	28 (15.3)	11 (39.3)	
*NG.*	No	182 (99.5)	36 (19.8)	0.620
	Yes	1 (0.5)	0 (0.0)	
*CT.*	No	175 (95.6)	35 (20.0)	0.602
	Yes	8 (4.4)	1 (12.8)	
Ureaplasma Urealyticum				
Uuu	No	175 (95.6)	34 (19.4)	0.698
	Yes	8 (4.4)	2 (25.0)	
Uup1	No	183 (100.0)	36 (19.7)	NA
	Yes	0 (0.0)		
Uup3	No	183 (100.0)	36 (19.7)	NA
	Yes	0 (0.0)	NA	
Uup6	No	183 (100.0)	36 (19.7)	NA
	Yes	0 (0.0)	NA	
Uup14	No	183 (100.0)	36 (19.7)	NA
	Yes	0 (0.0)	NA	
HSV-2	No	181 (98.9)	36 (19.9)	0.482
	Yes	2 (1.1)	0 (0.0)	
Plasma syphilis^a^	No	161 (88.0)	27 (16.8)	0.008
	Yes	22 (12.0)	9 (40.9)	
Rapid test of Hepatitis				
HAV-IgM	No	181 (98.9)	36 (19.8)	0.210
	Yes	2 (1.1)	0 (0.0)	
HBsAg	No	177 (96.7)	34 (19.2)	0.392
	Yes	6 (3.3)	2 (33.3)	
HCV-Ab	No	182 (99.5)	36 (19.8)	0.620
	Yes	1 (0.5)	0 (0.0)	

^a^, both RPR and TPPA positive testing results were regarded as currently infected with syphilis. *Uu ureaplasma urealyticum, Up Ureaplasma parvum*

The prevalence of HIV among subjects showed a dose-effect trend with number of STIs: for participants co-infected with 0, 1, 2, and 3 STIs, the prevalence of HIV was 11.2% (12/107), 29.0% (18/62), 38.5% (5/13) and 100% (1/1), respectively (trend Chi-square = 14.610, *P* = 0.001).

### Correlates of HIV infection

Chi-square test showed that age (*P* = 0.003), monthly income(*P* = 0.076), the main routes of seeking sexual partners (*P* = 0.015), number of casual male sexual partners (*P* = 0.123), Mycoplasma hominis infection (*P* = 0.033), Mycoplasma genitalium infection (*P* = 0.005), and Syphilis infection (*P* = 0.008) were significantly associated or marginally associated with HIV infection (Tables 1 and 2).

Multivariable logistic regression analysis showed that the use of partner-seeking apps (aOR = 2.6, 95%CI, 1.2–5.8, *P* = 0.017), Syphilis infection (aOR = 3.1, 95%CI, 1.2–8.0, *P* = 0.023), Mg infection (aOR = 3.2, 95%CI, 1.3–7.7, *P* = 0.012) and Mh infection (aOR = 2.1, 95%CI, 1.2–13.4, *P* = 0.020) were significantly associated with HIV infection (Table 4).

**Table 4:** Multivariable analysis of correlates of HIV infection in MSM attendants in Shenyang, China (*N* = 183)

Variables	aOR (95% CI)^a^	*P*-value
Use of partner-seeking apps	2.6(1.2–5.8)	0.017
Mycoplasma genitalium infection	3.2(1.3–7.7)	0.012
Mycoplasma hominis infection	4.1(1.2–13.4)	0.020
Syphilis infection	3.1(1.2–8.0)	0.023

^a^, adjusted for age, education and monthly economic income

## Discussion

We performed a cross-sectional study in Shenyang, China to determine correlates of HIV infection among MSM. We found that apps have become the predominant route of seeking male sexual partners and was independently associated with HIV infection. There was high prevalence of Mg, Mh and Syphilis infection, and infected MSM participants also had higher HIV prevalence. The prevalence of HIV showed a significant increasing trend with increasing number of STIs in MSM participants.

The prevalence of HIV among MSM in Shenyang was found to be more than 2.5 times the national average (19.7% vs. 7.75%) [22], and similar to prevalence estimates in the USA (14.5%), Canada (14.9%) and Australia (18.3%) [23]. This highlights the urgent need for comprehensive interventions to address risk factors for HIV among MSM. Although pre-exposure prophylaxis (PrEP) has been recommended by World Health Organization since 2014 to prevent HIV for high-risk populations, PrEP is currently not available for MSM in China. There are ongoing pilot studies evaluating the acceptability, adherence and even HIV risk compensation behavior of PrEP, which may help establish national guidelines to prevent HIV.

We found that the proportion of MSM who mainly sought out male sexual partners using apps was 51.4%, higher than a prior report of 43.3% from 2009 [24]. Further, MSM who mainly sought out male sexual partners using apps had 2.6 times the risk of HIV infection than MSM who mainly sought partners through other methods. This is consistent with results from two recent longitudinal cohort studies in MSM [3, 4], but differs from a recent Meta-analysis [5]. Our study results indicate that more effort should be dedicated to surveillance of MSM who seek sexual partners through apps and adaption of HIV prevention measures onto social networking mobile app platforms. One recent innovative intervention was implemented through an app popular among Chinese MSM to promote HIV testing and condom use [25, 26].

We simultaneously measured the prevalence of ten important STIs, including Mh, Mg, NG, and CT by using a novel multiple PCR (M-PCR) testing method [27]. This method may have important applications in STIs screening. In general, we found that the prevalence of HIV in MSM participants showed a significant dose-response trend with increased number of STIs. This further highlights the need for STI screening as a part of comprehensive HIV prevention.

In particular, we found that rectal infection with Mg was 15.3%, almost three times higher than rates reported from Shenzhen (5.4%) in 2014 [17], Italy (4.8%) [20] and Australia (8.9%) [18]. The prevalence of rectal Mg in HIV-positive MSM in this study was 4.8 times that reported in the US (30.6% vs. 6.4%) [21]. Additionally, we found that Mg infection was associated with a 3.2 times higher risk of HIV. Coinfection with Mg may cause mucosal disruption and inflammation that increases susceptibility to HIV [28, 29]. This result suggests that it is important to test and treat for Mg in Chinese MSM.

Similarly, we found a high prevalence of rectal Mh (7.1%), though lower than reports from MSM in Northern Ireland (24.3%) [19]. To our best knowledge, the relationship between Mh and HIV infection in MSM has not been previously reported. We found that Mh infection was associated with a 4.1 times risk of HIV infection, which was even greater than the association between HIV and Syphilis infection (aOR = 2.99) [30]. Our study suggests that Chinese MSM should also be screened for Mh. More research is required to investigate this association, and screening and treatment of Mh-positive MSM should be explored as a potential method of HIV prevention strategy.

We also noted the prevalence of HAV for the first time in an MSM population in mainland China. We found that it was higher than the HAV prevalence in the general population in US (1.1% vs. 1.3 per 100,000 population), but lower than that of MSM in the US (3.9%) [31]. Although the prevalence of HAV in our study appears to be lower than the prevalence reports from MSM in Korea (37.3%) and Bangkok (32.4%), but these two studies both measured HAV-IgG and not HAV-IgM, and may not be comparable with our result. Our result highlights the importance of screening for HAV and HAV vaccination among China MSM. China has had a free HBV vaccination program since 1992, but only implemented a free HAV vaccination program in 2008 [32], so Chinese MSM who are over 11 years or 27 years old are unlikely to have received the HAV vaccine and HBV vaccine, respectively. And there were scarcely plans or recommendations in China to provide HAV or HBV vaccination for MSM population. Hence our study indicated that the screening and vaccination against HAV and HBV among sexually active Chinese MSM should be considered, especially among the high risk individuals for HAV and HBV infection.

This study has several limitations. First, we only collected rectal swab specimens and did not collect urethral or oral swab specimens, so the overall prevalence of STIs is an underestimate. Secondly, we only used PCR testing for Mg, Mh, NG, CT, etc. and prevalence rates of STIs in this study may not be comparable with studies using culture or other diagnostic methods. Third, this is a cross-sectional design, which cannot determine the causal relationships between HIV and other STIs. Fourth, the study sample size is relatively small, which limits the statistical power of this study. Finally, MSM participants were recruited by convenience sampling from MSM seeking out HIV counseling and testing, and it is unclear whether this result is generalizable to the HIV prevalence of the general MSM population in Shenyang.

## Conclusions

There is increasing partner-seeking app usage among MSM, and app usage is correlated with risk of HIV infection. App platforms should be utilized to conduct HIV prevention interventions. Mg and Mh were both highly prevalent in our study, and both were independent risk factors for HIV infection. More effort should be dedicated to the screening, prevention and treatment of *Mycoplasma genitalium* and *Mycoplasma homini* infection, in order to curb the spread of HIV among MSM.

## Data Availability

The datasets used and/or analyzed during the current study are available from the corresponding author on reasonable request.

## Abbreviations

CI: Confidence interval
CT: *Chlamydia trachomatis*
Mg: *Mycoplasma genitalium*
Mh: *Mycoplasma hominis*
M-PCR: Multiple PCR
MSM: Men who have sex with men
NG: *Neisseria gonorrhoea*
ORs: Odds ratios
RPR: Rapid plasma reagent
STI: Sexually transmitted infection
TPPA: Treponema pallidum particle assay
Up: *Ureaplasma parvum*
Uu: *Ureaplasma urealyticum*
